# Peripheral hepatic sinusoidal obstruction syndrome due to oxaliplatin‐based chemotherapy

**DOI:** 10.1002/ccr3.1935

**Published:** 2019-01-09

**Authors:** Shinichiro Kobayashi, Sadayuki Okudaira, Kazuma Kobayashi, Kengo Kanetaka, Susumu Eguchi

**Affiliations:** ^1^ Department of Surgery Nagasaki University Graduate School of Biomedical Sciences Nagasaki Japan; ^2^ Okudaira Clinic Nagasaki Japan

**Keywords:** gadolinium‐ethoxybenzyl‐diethylenetriamine pentaacetic acid, gastric cancer, hepatic sinusoidal obstruction syndrome, magnetic resonance imaging, oxaliplatin

## Abstract

Hepatic sinusoidal obstruction syndrome during oxaliplatin‐based chemotherapy has been shown to be associated with severe steatohepatitis. Gadolinium‐ethoxybenzyl‐diethylenetriamine pentaacetic acid‐enhanced magnetic resonance imaging may identify various features of hepatic sinusoidal obstruction syndrome, even when the lesion cannot be differentiated from sinusoidal obstruction syndrome by other imaging tools.

## 1 CLINICAL IMAGE

The patient received oxaliplatin‐based chemotherapy for metastatic gastric cancer for 6 months. Abdominal computed tomography scan showed splenomegaly without any other abnormal finding. However, multi‐imaging modalities including gadolinium‐ethoxybenzyl‐diethylenetriamine pentaacetic acid‐enhanced magnetic resonance imaging showed the presence of cracked hypointensity of peripheral right hepatic lobule in the hepatobiliary phase.

**Figure 1:**
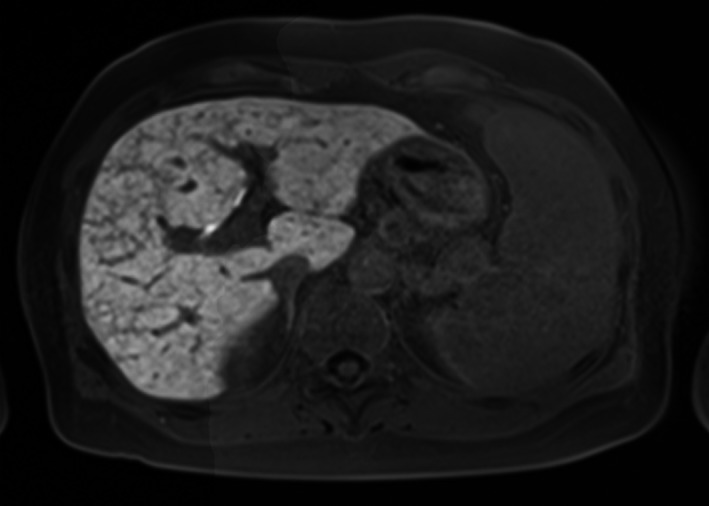
Gadolinium‐ethoxybenzyl‐diethylenetriamine pentaacetic acid‐enhanced magnetic resonance imaging showed the presence of cracked hypointensity of peripheral right hepatic lobule in the hepatobiliary phase

**Figure 2:**
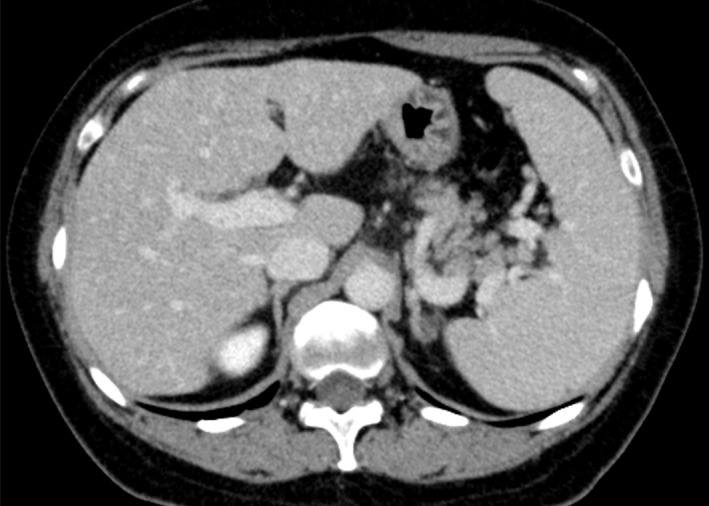
Abdominal computed tomography scan showed splenomegaly without any other abnormal finding

**Figure 3:**
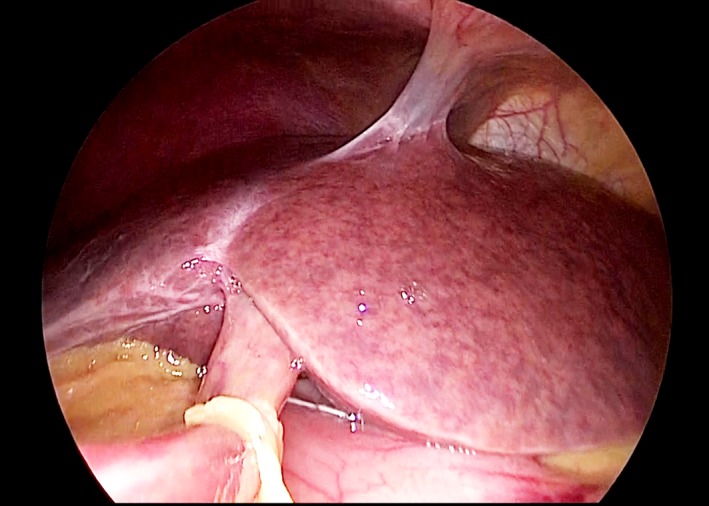
The blue network of fine crackles was found on the hepatic surface

**Figure 4:**
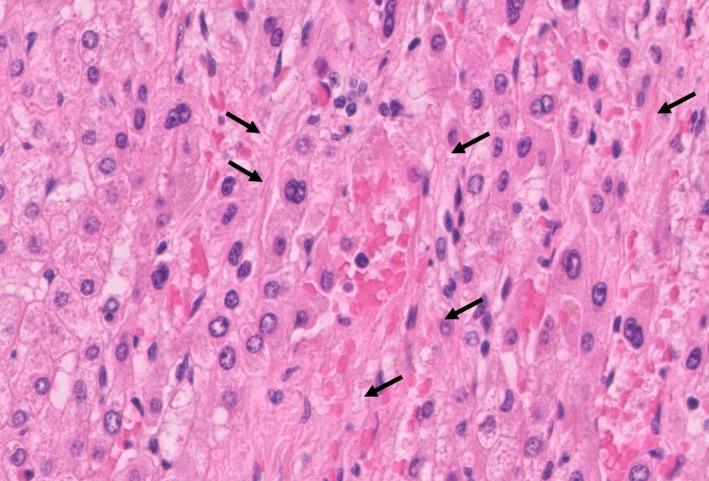
Some central veins displayed venous occlusion and recanalization in Hematoxylin‐Eosin staining

**Figure 5:**
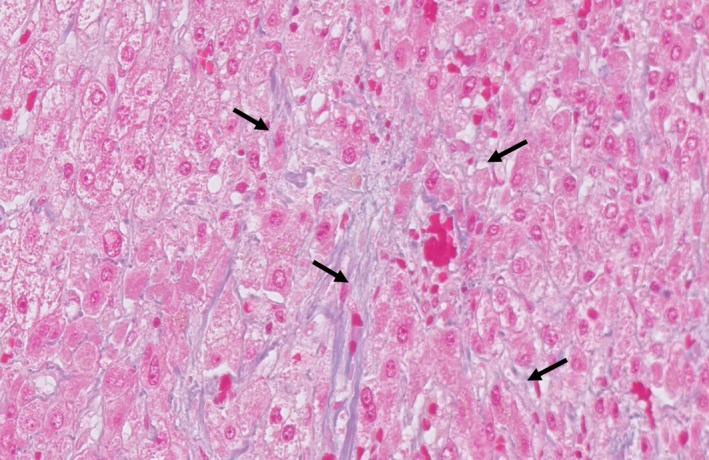
Diffuse sinusoidal fibrosis and congestion due to the disruption of the hepatocytic plate in AZAN staining

A 54‐year‐old woman with a history of chemotherapy against advanced gastric cancer with distant metastases in the liver and para‐aortic lymph nodes presented with acute‐onset epigastric pain and hematemesis. The patient received oxaliplatin‐based chemotherapy for metastatic gastric cancer for six months. We planned palliative gastrectomy against hematemesis from gastric cancer after evaluating distant metastatic lesions. Multi‐imaging modalities including gadolinium‐ethoxybenzyl‐diethylenetriamine pentaacetic acid ‐enhanced magnetic resonance imaging (EOB‐MRI) showed the presence of cracked hypointensity of peripheral right hepatic lobule in the hepatobiliary phase (Figure 1). Abdominal computed tomography scan showed splenomegaly without any other abnormal finding (Figure 2). Laboratory findings showed slightly elevated aspartate transaminase (38 IU/L), alanine aminotransferase (31 IU/L), alkaline phosphatase (341 IU/L), and gamma‐glutamyl transpeptidase (70 IU/L) levels. The patient received laparoscopic examination. The blue network of fine crackles was found on the hepatic surface (Figure 3) and incisional biopsy of the peripheral liver with palliative gastrectomy. Pathological findings showed diffuse sinusoidal fibrosis and congestion due to the disruption of the hepatocytic plate. Some central veins displayed venous occlusion and recanalization (Figure 4, Hematoxylin‐Eosin staining; Figure 5, AZAN staining). The patient was diagnosed as peripheral hepatic sinusoidal obstruction syndrome (SOS). She then continued chemotherapy without oxaliplatin to avoid severe steatohepatitis.^1^, ^2^ SOS may be considered one of the causes of newly developed hepatic lesions in patients with treated colorectal hepatic metastases.^3^, ^4^ Splenomegaly in CT scan may serve as a simple screening for identifying the patients.^5^ MRI scan is highly specific for the diagnosis of SOS.^6^, ^7^ In the patients with gastric cancer, MRI scan may also be useful for the diagnosis of SOS in several cases.^8^, ^9^ In summary, reticular hypointensity on hepatobiliary phase images of EOB‐MRI is an important indicator of SOS in the patients with gastric cancer.

## CONFLICT OF INTEREST

The authors declare no conflicts of interest in association with the present study.

## AUTHOR CONTRIBUTIONS

All of the authors have read and approved the manuscript. SK: collected, analyzed, and interpreted the patient disease data and edited the manuscript. Kazuma Kobayashi and Kengo Kanetaka: supervised the patient treatments and the research project. SO: pathologist and participated in the diagnosis of sinusoidal obstruction syndrome. SE: participated in the discussion and approved the final submission of this manuscript.

## ETHICAL APPROVAL

The publication of present study was in accordance with the ethical standards of our institution.

Consent for publication: Informed consent was obtained from the patient and her family in this report.

## References

[1] Vigano L , Rubbia‐Brandt L , De Rosa G , et al. nodular regenerative hyperplasia in patients undergoing liver resection for colorectal metastases after chemotherapy: risk factors, preoperative assessment and clinical impact. Ann Surg Oncol. 2015;22(13):4149‐4157. 25845431 10.1245/s10434-015-4533-0

[2] Soubrane O , Brouquet A , Zalinski S , et al. Predicting high grade lesions of sinusoidal obstruction syndrome related to oxaliplatin‐based chemotherapy for colorectal liver metastases: correlation with post‐hepatectomy outcome. Ann Surg. 2010;251(3):454‐460. 20160638 10.1097/SLA.0b013e3181c79403

[3] Seo AN , Kim H . Sinusoidal obstruction syndrome after oxaliplatin‐based chemotherapy. Clin Mol Hepatol. 2014;20(1):81‐84. 24757663 10.3350/cmh.2014.20.1.81 PMC3992335

[4] Choi JH , Won YW , Kim HS , Oh YH , Lim S , Kim HJ . Oxaliplatin‐induced sinusoidal obstruction syndrome mimicking metastatic colon cancer in the liver. Oncol Lett. 2016;11(4):2861‐2864. 27073565 10.3892/ol.2016.4286 PMC4812530

[5] Overman MJ , Maru DM , Charnsangavej C , et al. Oxaliplatin‐mediated increase in spleen size as a biomarker for the development of hepatic sinusoidal injury. J Clin Oncol. 2010;28(15):2549‐2555. 20406923 10.1200/JCO.2009.27.5701

[6] Ward J , Guthrie JA , Sheridan MB , et al. Sinusoidal obstructive syndrome diagnosed with superparamagnetic iron oxide‐enhanced magnetic resonance imaging in patients with chemotherapy‐treated colorectal liver metastases. J Clin Oncol. 2008;26(26):4304‐4310. 18779617 10.1200/JCO.2008.16.1893

[7] Shin NY , Kim MJ , Lim JS , et al. Accuracy of gadoxetic acid‐enhanced magnetic resonance imaging for the diagnosis of sinusoidal obstruction syndrome in patients with chemotherapy‐treated colorectal liver metastases. Eur Radiol. 2012;22(4):864‐871. 22108766 10.1007/s00330-011-2333-x

[8] Toi H , Miura Y , Shibasaki S , et al. Hepatic sinusoidal obstruction associated with S‐1 plus cisplatin chemotherapy for highly advanced gastric cancer with paraaortic lymph node metastases: report of a case. Clin J Gastroenterol. 2012;5(5):341‐346. 26181073 10.1007/s12328-012-0333-2

[9] Liu F , Cao X , Ye J , Pan X , Kan X , Song Y . Oxaliplatin‐induced hepatic sinusoidal obstruction syndrome in a patient with gastric cancer: A case report. Mol Clin Oncol. 2018;8(3):453‐456. 29468059 10.3892/mco.2017.1540 PMC5791411

